# Comparison of preoperative evaluation of malignant low-level biliary obstruction using plain magnetic resonance and coronal liver acquisition with volume acceleration technique alone and in combination

**DOI:** 10.1186/s40001-015-0188-3

**Published:** 2015-11-25

**Authors:** Nana Sun, Qing Xu, Xisheng Liu, Wei Liu, Jianwei Wang

**Affiliations:** Department of Radiology, the First Affiliated Hospital of Nanjing Medical University, Nanjing, 210029 Jiangsu China

**Keywords:** Bile duct obstruction, Ampulla of vater, Duodenal neoplasms, Pancreatic neoplasms, Magnetic resonance imaging, Coronal liver acquisition with volume acceleration technique

## Abstract

**Background:**

To evaluate the clinical value of plain magnetic resonance (MR) imaging (including magnetic resonance cholangiopancreatography, MRCP) and coronal liver acquisition with volume acceleration (LAVA) technique in the diagnosis and preoperative assessment of malignant low-level biliary obstruction.

**Methods:**

Forty-one patients with confirmed malignant low-level biliary obstruction were examined by plain MR, MRCP and coronal LAVA techniques. Group 1, plain MR (including MRCP); group 2, coronal LAVA; group 3, plain MR and coronal LAVA. Assessments included positioning, qualitative diagnosis and preoperative evaluation. The results were compared with pathological, endoscopic retrograde cholangiopancreatography or percutaneous transhepatic cholangiography results.

**Results:**

There were 14 pancreatic adenocarcinoma, 12 distal common bile duct carcinoma, 10 ampullary carcinoma, and 5 duodenal carcinoma cases. There was no significant difference in accuracy of the three groups’ positioning diagnoses, 87.8, 90.2, and 92.7 %, respectively. The accuracy of the qualitative diagnoses was lower in group 1 at 78.0 %, but not significantly different in groups 2 and 3 at 92.7 and 95.1 %, respectively (*P* = 0.031, and 0.039, group 1 vs groups 2 and 3, respectively). Thirty-three patients underwent open surgery. There were 19 adjacent organ involvements, 9 vascular involvements, 13 lymph node metastases and 6 liver metastases. 22 patients were verified surgically and histologically for resectable lesions. Plain MR with coronal LAVA imaging showed 85.4 % accuracy, 90.9 % sensitivity, 78.9 % specificity, 83.3 % positive and 88.2 % negative predictive value for resectability.

**Conclusions:**

Plain MR and coronal LAVA techniques are potential noninvasive tools for diagnosis and preoperative assessment of malignant low-level biliary obstruction.

**Electronic supplementary material:**

The online version of this article (doi:10.1186/s40001-015-0188-3) contains supplementary material, which is available to authorized users.

## Background

Malignant low-level biliary obstructions comprise carcinomas of the ampulla, distal common bile duct (CBD), pancreas, and duodenum. Their clinical features and anatomic locations are similar, as are the therapeutic approaches [1]. However, their 5-year survival rate and long-term outcomes vary [2–4]. Pancreatic carcinoma has the worst prognosis with 5-year survival rates around 5 % [5, 6]; if bile duct and ampullary carcinoma can be diagnosed early and treated promptly, the prognosis will be good and the 5-year survival rate can reach 40–50 % [3, 7–9]. Patients with ampullary or duodenal carcinoma have better 5-year survival rates than those with bile duct or pancreatic carcinoma [10, 11]. When diagnosed, 22–71 % of periampullary duodenal carcinomas are accompanied by lymph node metastasis, but even so the 5-year survival rate can reach 40–50 % [4, 5]. Obviously, it is essential to increase the surgical resectability and survival rate of patients.

Surgical resection has been the mainstay of curative treatment for malignant low-level biliary obstruction. In comparison to pancreatic head carcinoma, distal bile duct carcinoma is more resectable and less frequently demonstrates tumor spread to adjacent lymph nodes or the resection margin microscopically [12, 13]. For this reason, aggressive resection is indicated in ampullary or duodenal carcinoma [2].

It is difficult, but necessary, to attempt to discern diagnosis based on results of preoperative imaging. Ultrasound (US), computed tomography (CT), endoscopic retrograde cholangiopancreatography (ERCP) and magnetic resonance cholangiopancreatography (MRCP) are widely used for preoperative evaluation because of their availability and non-invasiveness. MRCP is one of the fastest examinations for the lesions of bile duct obstruction. With the advancement of magnetic resonance (MR) techniques, application of the liver acquisition with volume acceleration (LAVA) technique has provided us with a new method for making more accurate diagnosis of malignant low biliary obstruction [14], and has become a reliable basis for imaging diagnosis.

Both MR and the coronal LAVA techniques should be powerful tools in diagnosing malignant low-level biliary obstructions. This study investigated the diagnostic value of plain MR, MRCP and the coronal LAVA techniques in the patients with malignant low-level biliary obstruction and evaluated their effectiveness when used in isolation and in conjunction.

## Methods

### Patients

Between September 2012 and November 2013, 53 patients (29 men, 24 women; age range, 31–92 years; average age, 65 years) evaluated in our hospital that were suspected on a medical history and by previous imaging examination using different methods from those in this study (including ultrasonography, US, or contrast material-enhanced computed tomography, CT) of having a malignant low-level biliary obstruction, were considered for enrollment on this prospective study. The inclusion criteria were: adequate MR imaging quality for analysis, and surgery, endoscopic retrograde cholangiopancreatography (ERCP), or percutaneous transhepatic cholangiography (PTC) performed within 2 weeks of the MR examination in our hospital that confirmed malignant low-level biliary obstruction. Patients were excluded if they had a known hypersensitivity to gadopentetate dimeglumine, they did not have a confirmed malignant low-level biliary obstruction, or did not consent.

The patients gave written informed consent for their inclusion in this prospective study. The study was approved by the Ethics committee of our hospital.

### MRI scan sequences and parameters

All patients underwent plain MR, MRCP and coronal LAVA sequence using a 1.5-T MR system (Signa Excite HDx, General Electric Healthcare) and a torso phased array coil. Before the examination, patients were asked to fast 4–6 h before imaging, and coached in the required breathing and holding their breath technique. Patients received an intramuscular injection of anisodamine 10–20 mg half an hour before the examination, and were imaged in the supine position and respiratory-gated. A 22-gauge intravenous catheter was placed in an arm vein and connected to an MR compatible power injector. Patients underwent MRCP sequences first, and then plain MR and coronal LAVA sequences after drinking 800–1000 ml water. The parameters of the MR scan sequences are shown in Table 1. MRCP sequences included 2D thick slab single-shot fast spin-echo (FSE) MRCP and coronal 3D fast-recovery FSE MRCP with respiratory-gated, triggered at exhale, and thin multi-plane collection. The plain MR imaging sequences included axial breath-hold FSE T1-weighted imaging, FSE T2-weighted imaging, and coronal FIESTA sequence. Contrast-enhanced dynamic scanning with LAVA sequence was scanned by coronal plane or oblique coronal plane along the distal common bile duct and the ampullary structure. To decrease the angle deviation, two doctors decided the scanning line of the oblique LAVA sequence together. All patients received gadolinium contrast material (dose range 0.2 mmol/kg) at a flow rate of 2–3 ml/s followed by bolus of 20 ml physiologic saline solution. Coronal LAVA sequence was started at 15, 40 and 80 s after initialization of contrast material injection to obtain enhanced images corresponding to the early arterial, late arterial and portal venous phases, respectively. Acquisition of LAVA data for each phase was finished during a single breath-hold at the end of expiration (mean time 17 s). A coronal or oblique coronal scanning (3 min) and an axial scanning (4–5 min) were performed to acquire images of the delayed phase.

**Table 1:** MR scan sequences parameters

Protocol angulation	T1WI axial	T2WI axial	FIESTA coronal	2D MRCP oblique coronal	3D MRCP coronal	LAVA (oblique) coronal/axial multiphase
Sequence	FSE	FSE	FIESTA	SSFSE	FRFSE	LAVA
TR (ms)	400	6000.0	3.5	6000	3333	3.8
TE (ms)	8.0	85.008	1.5	798.3	1070.3	1.8
FOV (mm)	420	420	350	320	380	400
Matrix	320 × 256	288 × 224	224 × 224	384 × 288	320 × 256	288 × 200
No. of slices	20	20	20	6	113	84
Thickness (mm)	6	6	6	60.0	1.8	3
Voxel (mm)	1.3 × 1.6 × 6	1.5 × 1.9 × 6	1.6 × 1.6 × 6	0.8 × 1.1 × 60	1.2 × 1.5 × 1.8	1.4 × 2 × 3
Space (mm)	2.0	2.0	1.0	0.0	−0.90	−1.50
Bandwidth (kHz)	31.2	41.7	125	31.2	41.7	62.5
NEX	2.00	2.00	1.00	0.56	0.38	0.72
Respiratory	Breath-hold	Trigger	Trigger	Breath-hold	Trigger	Breath-hold

*MR* magnetic resonance, *MRCP* magnetic resonance cholangiopancreatography, *LAVA* liver acquisition with volume acceleration, *FIESTA* fast imaging employing steady-state acquisition, *FSE* fast spin-echo, *SSFSE* single shot fast spin-echo, *FRFSE* fast relaxation fast spin-echo, *TR* time of repeat, *TE* time of echo, *FOV* field of view, *NSA* number of signal average, *NEX* number of excitations

### Image analysis

Adequate MR imaging quality for analysis was required. Image quality was measured using a four-category scale as follows: nondiagnostic, anatomic structures not visible; poor diagnostic quality, anatomic structures partially visible; adequate diagnostic quality, anatomic structures mainly visible; and excellent diagnostic quality, excellent visualization of anatomic structures [15]. Images that did not exhibit adequate and excellent quality were removed. Image post-processing and analysis was performed using the advantage work-station 4.4 (AW4.4, GE healthcare, Fairfield, CT, USA). The source images were 3D reconstructed by using the maximum intensity projection (MIP) algorithm and volume-rendering (VR) functions in the workstation, thereby allowing the reconstructed 3D MRCP images to be rotated in any direction. We divided all MR images into three groups: group 1, plain MR images (including MRCP); group 2, coronal LAVA images (including axial delayed phase); group 3, plain MR with coronal LAVA images. The images from the three groups were viewed and analyzed together by two senior radiologists with more than 5 years of combined experience in abdominal MR diagnosis. Two radiologists read each group over at least 2 weeks interval, without knowing the clinical data, pathological information or the other images. Observation was focused on four aspects: (1) positioning diagnosis; (2) qualitative diagnosis: dilatation of the biliary and pancreatic ducts; the tumor morphological type, enhancement pattern; (3) infiltration extent of adjacent organ and vessels, lymph nodes, liver and distant metastases; (4) the prospective assessment of surgical resectability. The signs of an unresectable tumor were major abdominal organs (for example the bile duct, pancreas, duodenum or ampulla), or vascular infiltration (superior mesenteric artery (SMA), superior mesenteric vein (SMV) or portal vein (PV)), lymph nodes, liver or distant metastases. If none of these criteria were found, the tumor was regarded as resectable.

### Statistical analysis

The data from ERCP, PTC, surgical or pathological results of all cases were considered the final diagnoses. The MR diagnoses of three groups were compared to the final diagnoses in terms of the positioning diagnosis and qualitative diagnosis, and the diagnostic accuracy was calculated. All data were analyzed using the SPSS 13.0 (SPSS Inc. Chicago, IL, USA) statistical package. The McNemar test was used to pairwise comparison of three groups. If the data were insufficient, the Fisher exact test was used. A *P* value of <0.05 was used as the statistical significance level of the observed differences.

## Results and discussion

### Clinical and pathological results

MR images in the majority of patients exhibited adequate and excellent quality. There were seven patients excluded because of poor diagnostic quality. Among the remaining 46 patients, open surgery (*n* = 33), ERCP (*n* = 6), and PTC (*n* = 5) were performed. Two patients were examined at follow-up evaluation, with no evidence of progression of distal common bile duct lesions after 3, 6, and 12 months. Finally, 41 patients were enrolled into the study, including 14 cases of pancreatic adenocarcinoma, 12 cases of distal common bile duct carcinoma, 10 cases of ampullary carcinoma, and 5 cases of periampullary duodenal carcinoma. The other excluded five patients were 3 cases of cholangitis, including one case of common bile duct stenosis due to autoimmune pancreatitis; 1 case of pancreatic cystadenoma; 1 case of duodenal villous tubular adenoma.

### Tumor diagnosis and analysis

Table 2 gives the blinded readers’ final diagnoses for groups of malignant low-level biliary obstruction. In 41 patients with malignant low-level biliary obstruction, the positioning diagnostic accuracy of plain MR images (including MRCP), coronal LAVA images, and plain MR with coronal LAVA images was 87.8, 90.2, and 92.7 %, respectively. And there was no statistical difference among these three groups. The qualitative diagnostic accuracy of the three groups was 78.0, 92.7, and 95.1 %, respectively. The qualitative diagnostic accuracy of group 1 was lower than those of the other two groups (*P* = 0.031, and 0.039, respectively), and there was no statistical difference between group 2 and group 3. The positioning misdiagnoses and qualitative misdiagnoses of malignant low-level biliary obstruction are shown in Tables 3 and 4, respectively.

**Table 2:** Diagnostic accuracy of plain MR images (including MRCP), coronal LAVA images (including axial delayed phase), and plain MR with coronal LAVA images

Diseases	Number of cases	Positioning diagnosis			Qualitative diagnosis		
		Plain MR	Coronal LAVA	Plain MR with coronal LAVA	Plain MR	Coronal LAVA	Plain MR with coronal LAVA
Pancreatic adenocarcinoma	14	13	12	13	12	13	13
Distal common bile duct carcinoma	12	11	11	11	9	11	11
Ampullary carcinoma	10	8	9	9	7	9	10
Periampullary duodenal carcinoma	5	4	5	5	4	5	5
Total	41	36	37	38	32	38	39
Diagnostic accuracy		87.8 %	90.2 %	92.7 %	78.0 %	92.7 %	95.1 %

*MR* magnetic resonance, *MRCP* magnetic resonance cholangiopancreatography, *LAVA* liver acquisition with volume acceleration

**Table 3:** Positioning misdiagnosis of malignant low level biliary obstruction

Location of lesions	Plain MR		Coronal LAVA		Plain MR with coronal LAVA	
	Number	Misdiagnosis	Number	Misdiagnosis	Number	Misdiagnosis
Pancreas	1	Distal CBD	2	Distal CBD; Ampulla	1	Distal CBD
Distal CBD	1	Pancreatic head	1	Ampulla	1	Pancreatic head
Ampulla	2	Duodenum papilla; Distal CBD	1	Distal CBD	1	Distal CBD
Periampullary duodenum	1	Ampulla	–	–	–	–
Total	5	–	4	–	3	–

*MR* magnetic resonance, *MRCP* magnetic resonance cholangiopancreatography, *LAVA* liver acquisition with volume acceleration, *CBD* common bile duct

**Table 4:** Qualitative misdiagnosis of malignant low level biliary obstruction

Location of lesions	Plain MR		Coronal LAVA		Plain MR with coronal LAVA	
	Number	Misdiagnosis	Number	Misdiagnosis	Number	Misdiagnosis
Pancreatic adenocarcinoma	2	Inflammatory lesion	1	Inflammatory lesion	1	Inflammatory lesion
Distal CBD carcinoma	3	Inflammatory lesion (*n* = 1); Probably stone (*n* = 2)	1	Inflammatory lesion	1	Inflammatory lesion
Ampullary carcinoma	3	Inflammatory lesion	1	Inflammatory lesion	–	–
Periampullary duodenal carcinoma	1	Inflammatory lesion	–	–	–	–
Total	9	–	3	–	2	–

*MR* magnetic resonance, *MRCP* magnetic resonance cholangiopancreatography, *LAVA* liver acquisition with volume acceleration, *CBD* common bile duct

**Fig. 1:**
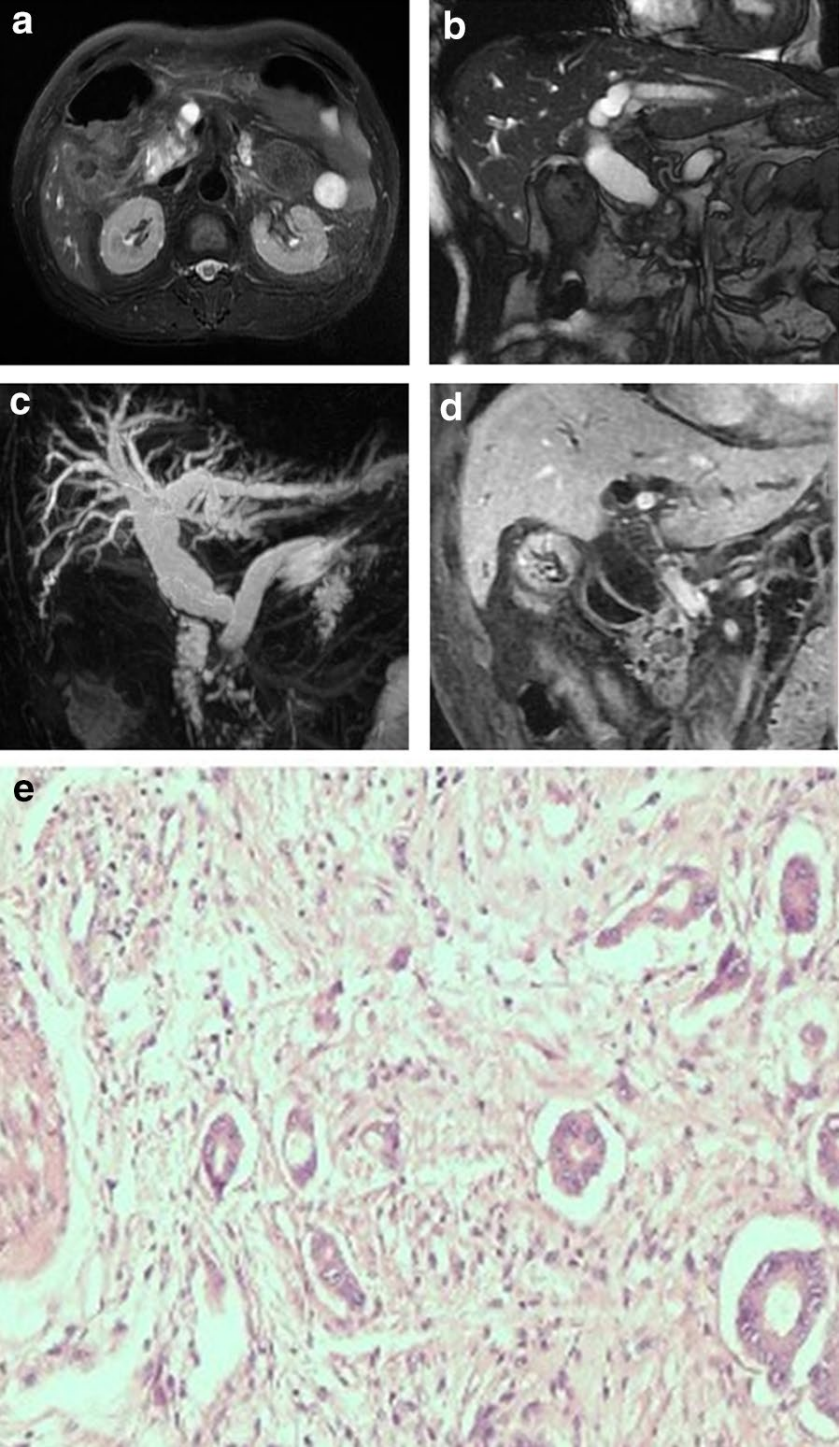
Pancreatic head carcinoma. Cholecystectomy was performed in a 68-year-old male patient with gallstones 9 years ago. **a** T2-weighted image (T2WI), high-intensity mass located in the pancreatic head; **b** fast imaging employing steady-state acquisition (FIESTA), the common bile duct (CBD) dilated and the distal segments of the bile duct were abruptly cut off; **c** magnetic resonance cholangiopancreatography (MRCP) showed dilation of the biliary tree and main pancreatic duct (PD). A congenital variability meant that the confluence of the hepatic duct and the cystic duct was at the distal bile duct. The distal segments of the bile duct and the pancreatic head segment of PD were abruptly cut off, forming the three-duct sign; **d** coronal enhanced liver acquisition with volume acceleration (LAVA) image, in the portal venous phase demonstrated a poorly enhanced mass (*narrow arrow*) around the distal common hepatic duct (CBD) and cystic duct; **e** photomicrograph (original magnification, ×400; hematoxylin–eosin stain)

**Fig. 2:**
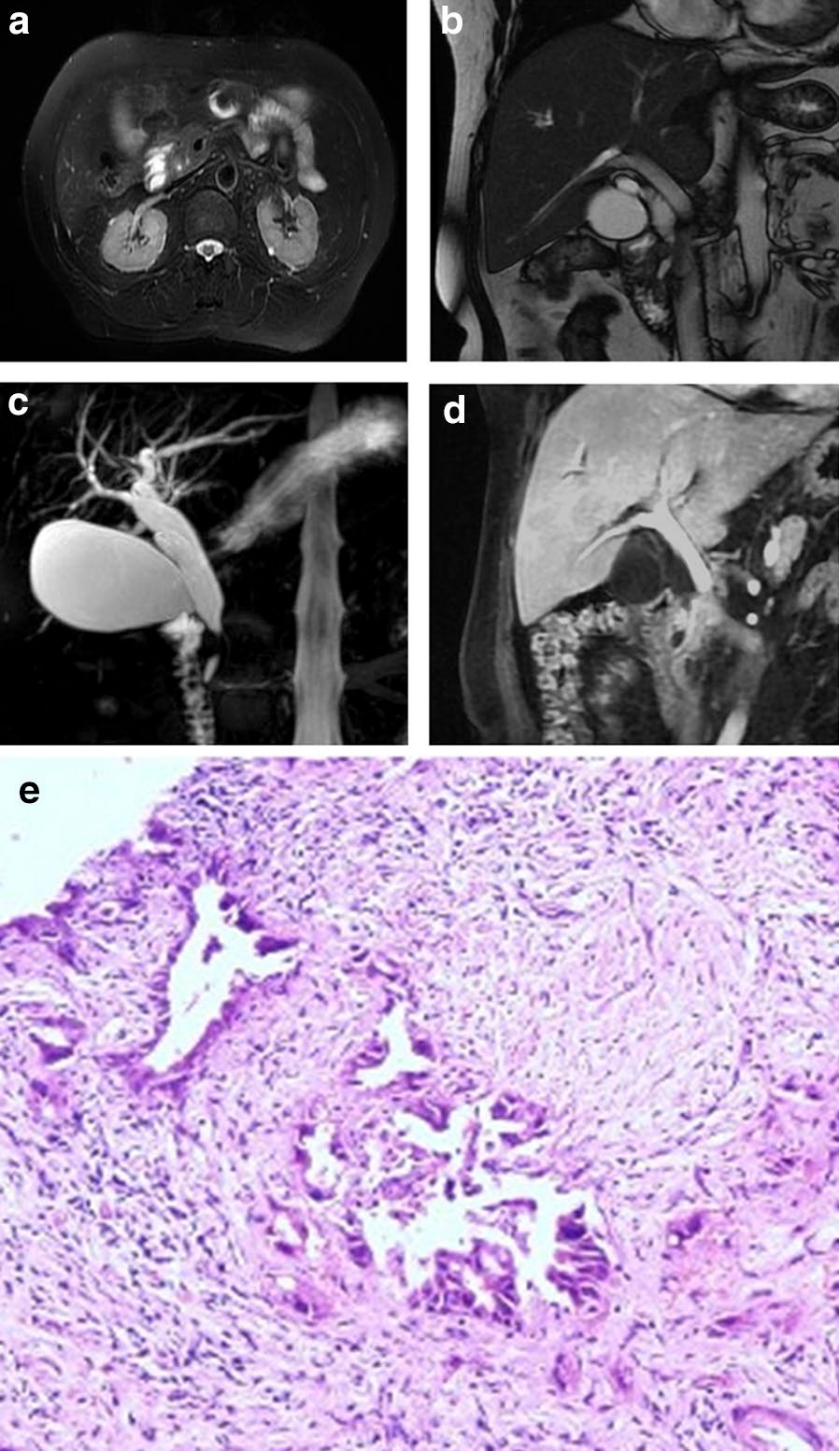
Distal common bile duct carcinoma. A 60-year-old male with distal common bile duct carcinoma. **a** T2WI, the wall of the distal CBD was thickened in the axial image; **b** an iso-intensity nodule could be seen at the distal segment of the CBD; **c** MRCP showed a filling defect in the dilated distal CBD without dilatation of the main pancreatic duct. The distal segment of the bile duct below the obstructive lesion was also seen. **d** Coronal enhanced LAVA image, in the portal venous phase showed enhancement of the thickened bile ductal wall and intraluminal papillary nodule. **e** Photomicrograph (original magnification, ×400; hematoxylin–eosin stain)

**Fig. 3:**
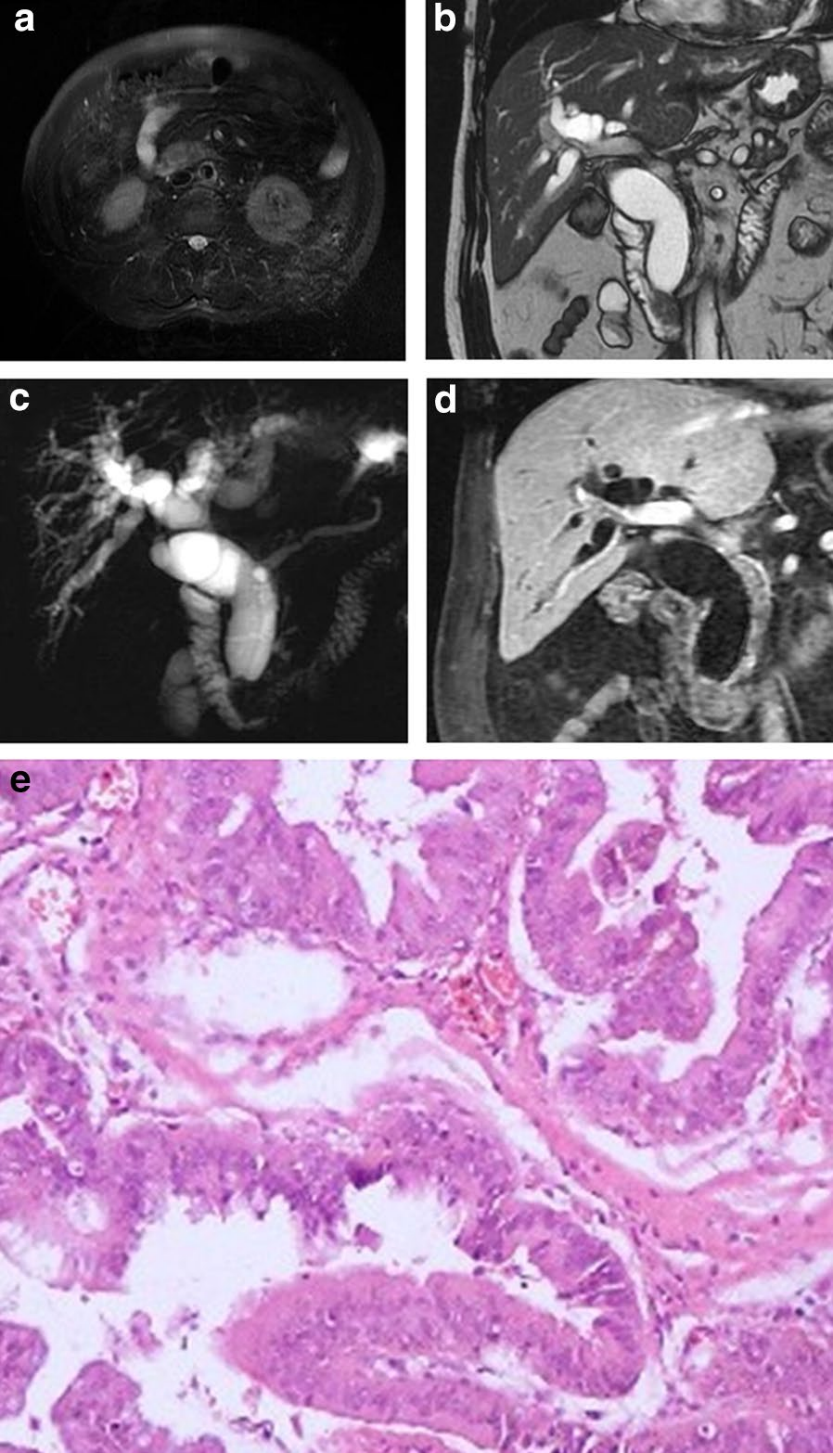
Ampullary carcinoma. Cholecystectomy was performed in a 60-year-old male patient with gallstones 7 years ago. **a** T2WI, iso-intensity mass could be seen at the ampullary area; **b** FIESTA, iso-intensity mass could be seen at the ampullary area; **c** MRCP showed a nodular filling defect at the distal end of the CBD (*arrow*) and markedly dilated CBD and pancreatic duct (PD); **d** coronal enhanced LAVA image, in the portal venous phase demonstrated moderate enhancement of the mass; **e** photomicrograph (original magnification, ×400; hematoxylin–eosin stain)

**Fig. 4:**
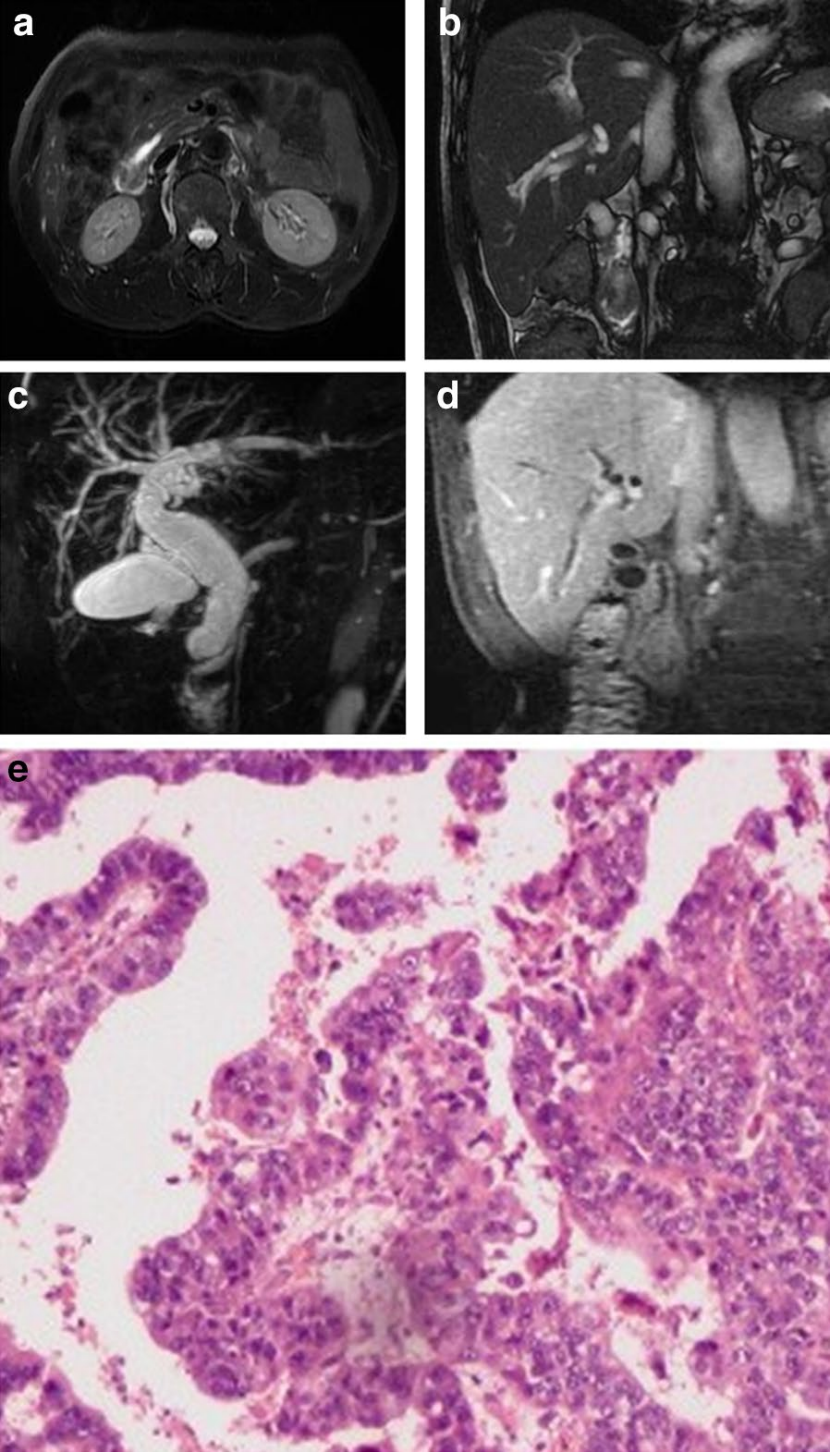
Duodenal papilla carcinoma. A 71-year-old male with duodenal papilla carcinoma. **a** T2WI, iso-intensity mass located in the descending duodenum and the proximal pancreatic duct dilated; **b** FIESTA, the oval mass showed slightly higher intensity; **c** MRCP image showed a nodular filling defect in the periampullary duodenal lumen (*arrow*) and markedly dilated CBD and PD; **d** coronal enhanced LAVA image, in the portal venous phase demonstrated mild enhancement of the mass; **e** photomicrograph (original magnification, ×400; hematoxylin–eosin stain)

**Fig. 5:**
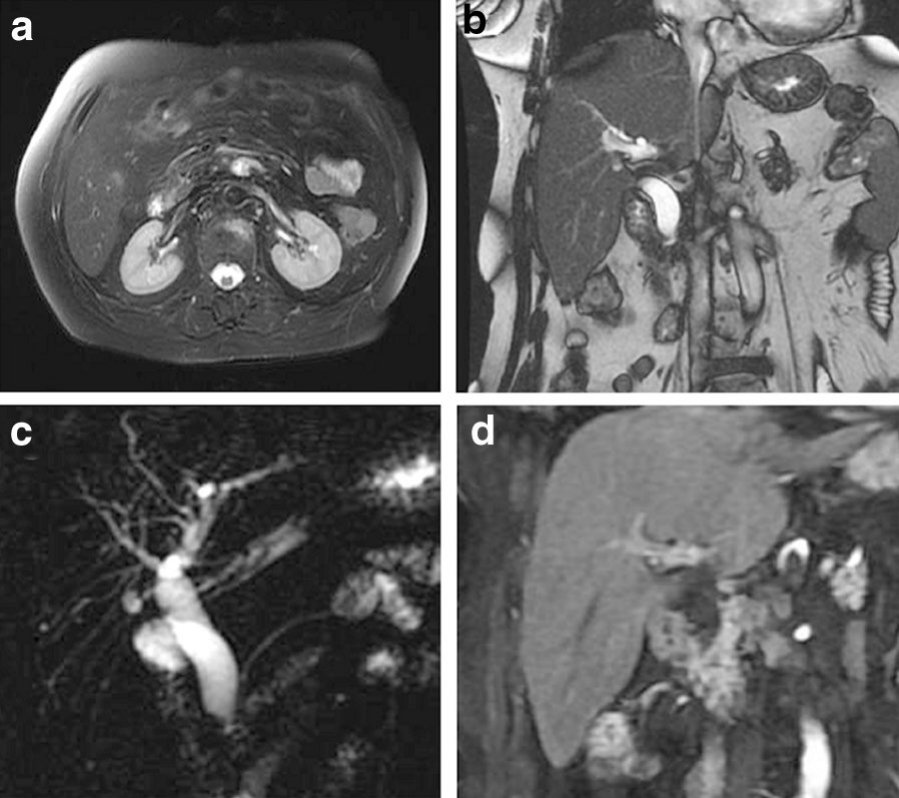
Distal common bile duct inflammatory stenosis. Cholecystectomy was performed in a 78-year-old female patient with gallstones 10 years ago. **a** T2WI, observation of the narrow segment of the CBD was not clear; **b** FIESTA, the distal CBD narrowed; **c** MRCP image showed distal CBD stenosis with biliary duct dilatation; **d** coronal enhanced LAVA image, in the arterial phase demonstrated linear homogeneous enhancement of the incrassate bile ductal walls

Details of the different diagnoses and the numbers of cases are shown in the Additional file 1 and examples are shown in Figs. 1, 2, 3, 4 and 5. These were pancreatic head adenocarcinoma shown in Fig. 1, distal common bile duct carcinoma shown in Fig. 2, ampullary carcinoma shown in Fig. 3, periampullary duodenal carcinoma shown in Fig. 4, and distal common bile duct inflammatory stenosis shown in Fig. 5.

### Assessment of the range of tumor invasion

**Fig. 6:**
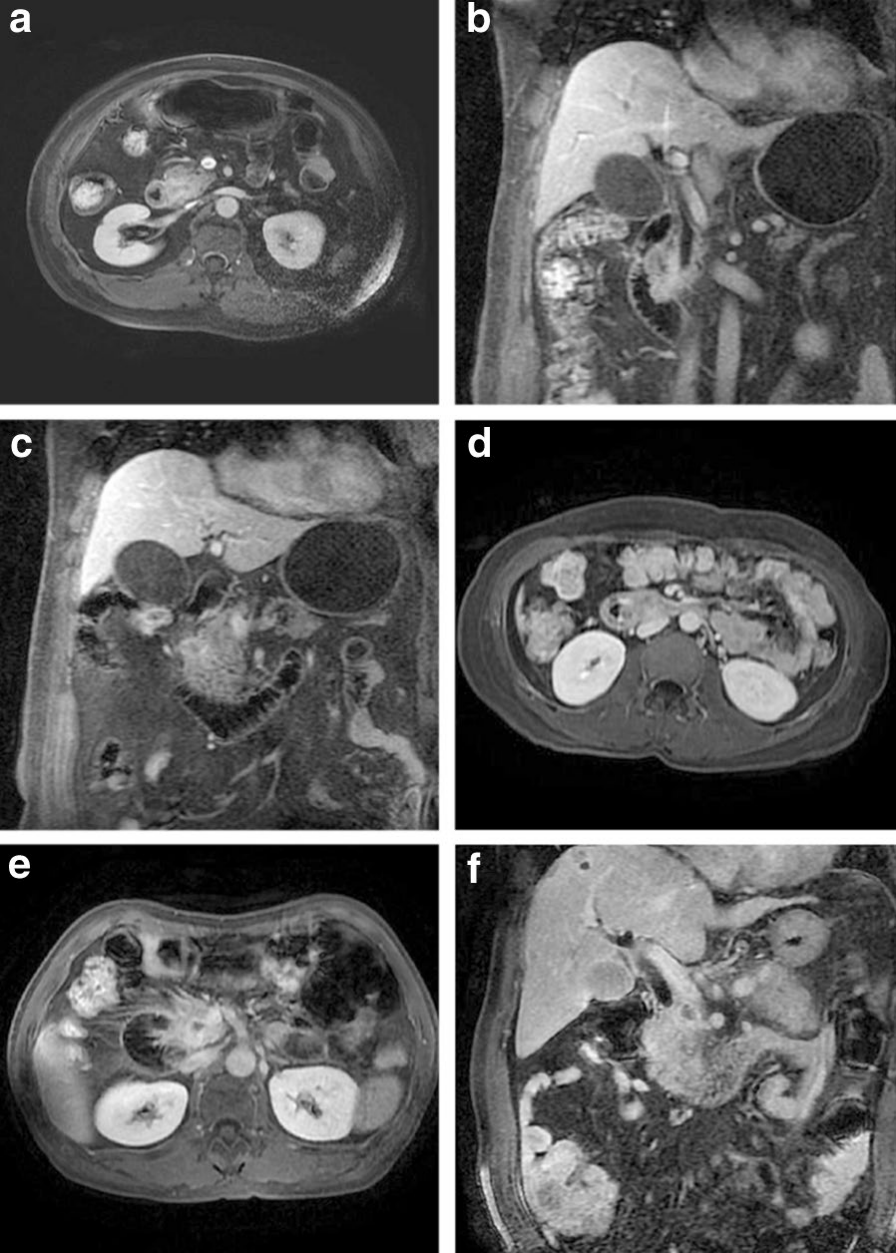
Examples of tumor invasion. Examples of invasive cancers **a**–**c**: a 69-year-old male with ampullary carcinoma. **a** A portion of pancreas and the descending duodenum were involved. **b** Coronal enhanced liver acquisition with volume acceleration (LAVA) showed descending duodenum invasion. **c** The coronal LAVA image showed pancreatic involvement. **d** A 48-year-old female with ampullary carcinoma, local tumor with unclear boundaries of the inferior vena cava, surgery confirmed the inferior vena cava was involved. *Figure* **e** male, common bile duct cancer, tumor invasion of the portal vein; **f** male, 67 years, liver metastases of pancreatic cancer

Thirty-three patients underwent open surgery, and the surgical and histopathological findings were considered as the final results. There were 19 adjacent organ involvements, 9 vascular involvements, 13 lymph node metastases and 6 liver metastases. Examples of invasive cancers are given in Fig. 6. Table 5 gives the comparison of plain MR with coronal LAVA imaging versus the final diagnosis in tumor invasion. Duodenal invasion in one ampullary carcinoma patient and ampulla invasion in one distal CBD carcinoma patient were missed. Pancreatic head carcinoma infiltration was overestimated in one patient. Vascular infiltration of the superior mesenteric vein (SMV) was missed in one patient and of the superior mesenteric artery (SMA) was missed in two patients. Vascular infiltration of the SMA was overestimated in one patient. In one patient, metastatic lymph nodes of normal size were missed. In two patients, enlarged abdominal lymph nodes diagnosed malignant on MR imaging were histologically proved to be chronic inflammation. An atypical hemangioma in liver was misdiagnosed as metastasis.

**Table 5:** Comparison of plain MR with coronal LAVA imaging versus final diagnosis in tumor invasion

	Accuracy (%)	Sensitivity (%)	Specificity (%)	Positive predictive value (%)	Negative predictive value (%)
Adjacent organ infiltration	91 (30/33)	89 (17/19)	93 (13/14)	94 (17/18)	87 (13/15)
Vascular infiltration	88 (29/33)	67 (6/9)	96 (23/24)	86 (6/7)	88 (23/26)
Lymph node metastases	91 (30/33)	92 (12/13)	90 (18/20)	86 (12/14)	95 (18/19)
Liver metastases	97 (32/33)	100 (6/6)	96 (26/27)	86 (6/7)	100 (26/26)

*MR* magnetic resonance, *LAVA* liver acquisition with volume acceleration

### Tumor resectability assessment

Of 41 patients with malignant low-level biliary obstruction, 22 patients were verified surgically and histologically that the lesions were resectable. The accuracy, sensitivity, specificity, positive and negative predictive value of plain MR with coronal LAVA imaging in assessing the resectability of malignant low-level biliary obstruction tumors were 85.4, 90.9, 78.9, 83.3, and 88.2 %, respectively (Table 6). Two misdiagnosed cases originally considered unresectable were caused by diagnosing the atypical hemangioma in the liver as metastasis and vascular infiltration of pancreatic head carcinoma. In four cases initially considered to be resectable (two pancreatic head carcinomas and two distal CBD carcinomas), adjacent organ and vascular infiltration were confirmed at surgery.

**Table 6:** Assessment of tumor resectability

Plain MR with coronal LAVA assessment	Final diagnosis		
	Unresectable	Resectable	Total
Unresectable (+ invasion/metastasis)	15	2^a^	17
Resectable (− invasion/metastasis)	4^b^	20	24
Total	19	22	41

*MR* magnetic resonance, *LAVA* liver acquisition with volume acceleration

^a^False-positive for invasion/metastasis

^b^False-negative for invasion/metastasis

## Discussion

Low-level biliary obstruction tumors may share clinical presentations, anatomic locations, and therapeutic approaches, but their long-term outcomes are varied [2–4]. Accurate diagnosis and determination of the tumor origin may affect treatment planning and predicting prognosis. The aims of this study were to evaluate the clinical value of plain MR and coronal LAVA techniques in diagnosing and preoperative assessing malignant low-level biliary obstruction diseases. We found that plain MR and the coronal LAVA techniques when used in isolation and together could achieve accuracy of positioning diagnoses of, 87.8 % for plain MR 90.2 % for coronal LAVA and 92.7 % for the two combined. The accuracy of the qualitative diagnoses was lower for plain MR at 78.0 %, but similar at 92.7 % for coronal LAVA, and 95.1 % for the combined methods. The low qualitative diagnostic accuracy for plain MR was probably because of the insufficient spatial resolution and of the plain MR sequences. Meanwhile, the slice thickness, the size and location of tumor, artifacts all influence qualitative diagnoses. The information of plain T1W and T2W images was not enough for improving the accuracy of qualitative diagnoses. The methods also proved useful for predicting tumor resectability as plain MR with coronal LAVA imaging showed 85.4 % accuracy, 90.9 % sensitivity, 78.9 % specificity, 83.3 % positive and 88.2 % negative predictive value.

The reason for misdiagnosis with coronal LAVA scan was often that it was more difficult to identify the origin of the tumor, when the tumor was large and had invaded the surrounding tissue. For example, the origin of a pancreatic carcinoma that had infiltrated the distal CBD was difficult to distinguish from a distal CBD carcinoma that had infiltrated the pancreatic head. Sometimes, ampullary carcinomas and distal CBD carcinomas were also difficult to differentially diagnose. Some cases in our study proved difficult to distinguish between mucosal enhancement of bile ducts due to tumor spreading or reactive inflammation on the coronal LAVA images.

3D MRCP can show the site and extent of the biliary tree obstruction clearly, and it has the advantage of providing positioning diagnosis of low-level biliary obstruction. MRCP relies mainly on morphological changes of pancreaticobiliary ducts to initially determine the nature of lesions. Neoplastic lesions may present as blunted, beak, or rat-tail forms of the distal bile duct and remarkably dilated bile ducts. The inflammatory narrow bile duct gradually tapers, and the edge is symmetrical and regular. Biliary tree dilatation is milder than that of neoplastic lesions. Filling defects in bile duct intraluminal probably suggest stones. MRCP can provide intuitive and reliable information about the pancreaticobiliary duct and is considered as a replacement for diagnostic ERCP [16]. However, MRCP images are heavily T2-weighted, highlighting water and lacking soft tissue contrast. So MRCP is not suitable for observing lesions and structure outside the lumen or for the qualitative diagnosis of low-level bile duct obstruction. In addition, the tapered area of the distal biliary and pancreatic ducts contains little or no fluid. As a result, it is difficult to reveal any small mass at the ampulla. Physiologic contraction of the sphincter of Oddi also makes it difficult to evaluate the periampullary area [2]. Fast imaging employing steady-state acquisition (FIESTA) sequence has very short repetition time (TR) and echo time (TE), fast imaging speed and high signal-to-noise ratio (SNR). However, FIESTA imaging displays the anatomical structures in general and it may easily miss shorter T2 lesions.

It is difficult for a conventional axial plain MR scan to differentially diagnose small masses and the morphological details of ampullary carcinoma, infiltrative common bile duct (CBD) carcinoma and CBD inflammatory stenosis because of the inherent limits of relatively thick sections and the interslice gap [17]. In these cases, there is an advantage in using the coronal LAVA sequence method for observation of small lesions and their enhanced features. In addition, stimulated by long-term inflammation in some patients with bile duct inflammatory stenosis, bile duct lesions may be malignant. It is therefore necessary to identify whether the lesions are benign or malignant using the enhanced LAVA sequence. However, there are also other imaging examinations including CT, diffusion-weighted MR imaging (DWI) and positron emission tomography (PET). MR has more advantages than CT because of its excellent soft tissue resolution, multiple-plane scanning and no radiation. DWI is helpful to diagnose some water molecules diffusion-limited lesions like malignant tumors. However, limitation of slice thickness and spatial resolution leads to misdiagnosis of some small local lesions. PET is not commonly used to diagnose low-level biliary obstruction. LAVA sequence has a fast scanning speed, high SNR, high soft tissue contrast and high sensitivity for showing lesions, and has more homogeneous fat suppression. Meanwhile it can improve the detection of small lesions, especially enhanced in the delayed phase [4]. For diagnosis of malignant low biliary obstruction, LAVA sequence can be scanned by coronal plane or oblique coronal plane along the common bile duct rather than the conventional axial plane, mainly based on the complex anatomical characteristics of the ampullary region. According to axial T2W images, the scanning position line of coronal plane or oblique coronal plane was selected as far as possible from the ligature of the distal CBD and ampullary showed clearly. The ampullary region is adjacent to the distal CBD, the pancreatic head and duodenum and it can be difficult to observe small lesions of ampullary region in axial planes. This can also dynamically observe the location and enhancement pattern of the lesions and the situation of the surrounding tissue infiltrated by malignant tumors. Furthermore, for coronal or oblique coronal imaging the patients should drink 800–1000 ml water and have an intramuscular injection of anisodamine (654-2) 10–20 mg before the scan to attenuate gastrointestinal peristalsis and make the duodenum hypotonic. This displays the ampullary regional lesions much better under the background of low signal water in the descending duodenum. In addition, reducing the field of view (FOV) and slice thickness, can not only shorten the scan time of each phase, but also aid observation of the local fine structure, which facilitates early detection of tumors. When the slice thickness is less than 3 mm the SNR significantly reduces making it difficult to meet the diagnostic needs. Although coronal LAVA images have advantages of displaying the CBD, ampulla and its surrounding tissues, when a comprehensive assessment of the lesions is demanded, it is necessary to combine the axial images to observe vascular invasion, lymph nodes metastasis, liver and other organs metastases. So axial delayed phase scanning can be performed 4–5 min after the contrast material has been injected.

Assessment of the range of tumor invasion is important for planning the appropriate treatment and predicting prognosis. In our study, direct invasion of adjacent organs was frequently observed. In pancreatic carcinoma, the tumor commonly invaded the distal CBD and the descending duodenum with regional lymph nodes metastases free, although in very rare cases of metastasis did occur. In contrast, other studies have reported pancreatic carcinomas tend to metastasize to regional lymph nodes in the early period [14, 15]. Ampullary carcinoma commonly invaded the descending duodenum with regional lymph nodes mostly metastases free in our study. Joo Hee Kim et al. [2] reported that lymphatic spread and perineural invasion were also infrequent in ampullary carcinoma. Pancreatic infiltration was commonly seen in the distal CBD carcinoma in this study. The enhanced MR images displayed the relationship of the tumor with vascular wall including superior mesenteric vein, superior mesenteric artery, portal vein and inferior vena cava. Our study showed the infiltrated vessels were frequently found in SMA and SMV. Vascular infiltration showed (a) the tumor surrounded blood vessels; (b) fat space around the vessels involved or disappeared; or (c) the vascular wall was irregular, vascular lumen was narrow or deformed. Similar to our study, pancreatic carcinoma has been shown to frequently infiltrate vessels in previous studies [6, 18, 19]. However, the sensitivity of vascular infiltration (67 %) appeared to be similar or lower (61.5–94 %) than in other studies [14, 15]. This low sensitivity might be because it was sometimes very difficult to identify whether the tumor had infiltrated or was very close to the vascular wall even with dynamic MR studies. Moving or breathing artifacts also interfered with the readers’ analysis of the images that the sensitivity of vascular infiltration could decrease. In our study, there was a case of SMA infiltration missed because of breathing artifact. Previous studies have regarded MR angiography as a good approach for the evaluation of vascular infiltration [15, 20, 21]. It is generally accepted that metastatic lymph nodes are larger than 1 cm. However, small lymph nodes less than 1 cm in diameter seen in the images were occasionally pathologically proved to be metastatic. In contrast, lymph nodes larger than 1 cm in diameter were sometimes pathologically proved to be inflammatory in other cases. However, in our study, the number of patients with these situations was low.

Another important assessment that these imaging evaluations need to perform is whether the tumor can be resected. The signs of unresectable tumor in this study were major abdominal organ or vascular infiltration, liver, and peritoneal or distant metastases. In our study, 53.7 % patients with malignant low-level biliary obstruction had resectable tumors, and the accuracy of plain MR with coronal LAVA imaging in assessing the resectability of malignant low-level biliary obstruction tumors was 85.4 %, that was similar to the values (88–90.4 %) of previous studies [14]. Our study showed that in four cases the degree of vascular and adjacent organ infiltration was underestimated.

There were three cases of cholangitis, including one case of common bile duct stenosis due to autoimmune pancreatitis in our study. Findings of the two cases of cholangitis did not change at reevaluation at 3, 6, and 12 months follow-up. The features of the case of autoimmune pancreatitis were diffuse pancreas enlargement and a terminal CBD stricture. After the patient took corticosteroids for 2 weeks, symptoms of obstructive jaundice significantly improved. Biliary inflammatory stricture is common in clinical works and is sometimes difficult to differentiate from bile duct carcinoma. Thus, we need to combine the patients’ medical history (history of biliary stones or inflammation), blood, tumor markers and long-term follow-up for comprehensive assessment.

This study has some limitations: we did not do the study of the angle deviation of the oblique coronal LAVA influencing on the diagnostic accuracy; we did not compare MR images with CT images, nor coronal scanning with axial scanning to fully evaluate the best imaging method for preoperative evaluation. The methods used in this study required the patients to perform uniform breathing and breath-hold during scanning. It was very important to train the patients in the breathing techniques before scanning, otherwise the resulting image was fuzzy and unfavorable for evaluation. It was difficult to determine the origin if the tumors were large and invading the surrounding tissues, such as pancreatic carcinoma invading the CBD and distal CBD carcinoma invading the pancreas. In the future, more research is needed to solve these problems.

In summary, MRCP can directly show the pancreaticobiliary tree, the location of biliary obstruction, and the degree of biliary dilatation. Coronal or oblique coronal LAVA scanning is helpful for displaying the distal CBD and ampullary region comprehensively and increasing the positioning and qualitative diagnostic accuracy, especially qualitative diagnoses of malignant low-level biliary obstruction. Combining axial images is necessary when tumor invasion and resectability are assessed.

## Additional file

10.1186/s40001-015-0188-3 Diagnosis results of low level biliary obstruction.

## Abbreviations

MR: magnetic resonance
LAVA: liver acquisition with volume acceleration
CBD: common bile duct
US: ultrasound
CT: computed tomography
ERCP: endoscopic retrograde cholangiopancreatography
MRCP: magnetic resonance cholangiopancreatography
FIESTA: fast imaging employing steady-state acquisition
SNR: signal-to-noise ratio
FOV: field of view
PTC: percutaneous transhepatic cholangiography
CEA: carcinoembryonic antigen
ALT: alanine aminotransferase
AST: aspartate aminotransferase
ALP: alkaline phosphatase
GCT: γ-glutamyltransferase
TB: total bilirubin
DB: direct bilirubin
IB: indirect bilirubin
FSE: fast spin-echo
MIP: maximum intensity projection
VR: volume-rendering
DWI: diffusion-weighted imaging
PET: positron emission tomography
SMA: superior mesenteric artery
SMV: superior mesenteric vein
PV: portal vein

## References

[1] Chan C, Herrera MF, de la Garza L, Quintanilla-Martinez L, Vargas-Vorackova F, Richaud-Patin Y, Llorente L, Uscanga L, Robles-Diaz G, Leon E (1995). Clinical behavior and prognostic factors of periampullary adenocarcinoma. Ann Surg.

[2] Kim JH, Kim MJ, Chung JJ, Lee WJ, Yoo HS, Lee JT (2002). Differential diagnosis of periampullary carcinomas at MR imaging. Radiographics.

[3] Chen CH, Tseng LJ, Yang CC, Yeh YH, Mo LR (2001). The accuracy of endoscopic ultrasound, endoscopic retrograde cholangiopancreatography, computed tomography, and transabdominal ultrasound in the detection and staging of primary ampullary tumors. Hepatogastroenterology.

[4] Chen WX, Xie QG, Zhang WF, Zhang X, Hu TT, Xu P, Gu ZY (2008). Multiple imaging techniques in the diagnosis of ampullary carcinoma. Hepatobiliary Pancreat Dis Int.

[5] Sarmiento JM, Nagomey DM, Sarr MG, Farnell MB (2001). Periampullary cancers: are there differences?. Surg Clin North Am.

[6] Prokesch RW, Chow LC, Beaulieu CF, Bammer R, Jeffrey RB (2002). Isoattenuating pancreatic adenocarcinoma at multi-detector row CT: secondary signs. Radiology.

[7] Huang WC, Sheng J, Chen SY, Lu JP (2011). Differentiation between pancreatic carcinoma and mass-forming chronic pancreatitis: usefulness of high b value diffusion-weighted imaging. J Dig Dis.

[8] Wang LF, Hingorani SR, Tuveson DA (2004). Detecting and diagnosing ampullary neoplasms. Cancer Biol Ther.

[9] Chung YE, Kim MJ, Park MS, Choi JY, Kim H, Kim SK, Lee M, Kim HJ, Choi JS, Song SY, Kim KW (2010). Differential features of pancreatobiliary- and intestinal-type ampullary carcinomas at MR imaging. Radiology.

[10] Pickleman J, Koelsch M, Chejfec G (1997). Node-positive duodenal carcinoma is curable. Arch Surg.

[11] Su CH, Shyr YM, Lui WY, P’Eng FK (1999). Factors affecting morbidity, mortality and survival after pancreaticoduodenectomy for carcinoma of the ampulla of Vater. Hepatogastroenterology.

[12] Fong Y, Blumgart LH, Lin E, Fortner JG, Brennan MF (1996). Outcome of treatment for distal bile duct cancer. Br J Surg.

[13] Yeo CJ, Cameron JL, Sohn TA, Lillemoe KD, Pitt HA, Talamini MA, Hruban RH, Ord SE, Sauter PK, Coleman J, et al. Six hundred fifty consecutive pancreaticoduodenectomies in the 1990s: pathology, complications, and outcomes. Ann Surg. 1997;226:248–57 **(discussion 257–260)**. 10.1097/00000658-199709000-00004 PMC1191017 9339931

[14] Zhong L, Li L, Yao QY (2005). Preoperative evaluation of pancreaticobiliary tumor using MR multi-imaging techniques. World J Gastroenterol.

[15] Lopez Hanninen E, Amthauer H, Hosten N, Ricke J, Bohmig M, Langrehr J, Hintze R, Neuhaus P, Wiedenmann B, Rosewicz S, Felix R. Prospective evaluation of pancreatic tumors: accuracy of MR imaging with MR cholangiopancreatography and MR angiography. Radiology. 2002;224:34–41. 10.1148/radiol.2241010798 12091659

[16] Irie H, Honda H, Shinozaki K, Yoshimitsu K, Aibe H, Nishie A, Nakayama T, Masuda K (2002). MR imaging of ampullary carcinomas. J Comput Assist Tomogr.

[17] Yin LL, Song B, Xu J, Li YC (2007). Hilar cholangiocarcinoma: preoperative evaluation with a three dimensional volumetric interpolated breath-hold examination magnetic resonance imaging sequence. Chin Med J (Engl).

[18] Lepanto L, Arzoumanian Y, Gianfelice D, Perreault P, Dagenais M, Lapointe R, Letourneau R, Roy A (2002). Helical CT with CT angiography in assessing periampullary neoplasms: identification of vascular invasion. Radiology.

[19] Arslan A, Buanes T, Geitung JT (2001). Pancreatic carcinoma: MR, MR angiography and dynamic helical CT in the evaluation of vascular invasion. Eur J Radiol.

[20] Sadick M, Diehl SJ, Lehmann KJ, Gaa J, Mockel R, Georgi M (2000). Evaluation of breath-hold contrast-enhanced 3D magnetic resonance angiography technique for imaging visceral abdominal arteries and veins. Invest Radiol.

[21] Catalano C, Pavone P, Laghi A, Panebianco V, Scipioni A, Fanelli F, Brillo R, Passariello R (1998). Pancreatic adenocarcinoma: combination of MR imaging, MR angiography and MR cholangiopancreatography for the diagnosis and assessment of resectability. Eur Radiol.

