# Supplementary material for: Comparison of preoperative evaluation of malignant low-level biliary obstruction using plain magnetic resonance and coronal liver acquisition with volume acceleration technique alone and in combination
Source: Eur J Med Res. 2015 Nov 25;20:92. doi: 10.1186/s40001-015-0188-3 (PMC4660785; doi:10.1186/s40001-015-0188-3)
Supplement: Supplementary file 1 — 10.1186/s40001-015-0188-3 Diagnosis results of low level biliary obstruction. [file 40001_2015_188_MOESM1_ESM.docx]

**SUPPLEMENTARY INFORMATION**

Details of the different diagnoses are presented below.

***Pancreatic head adenocarcinoma***

Large pancreatic head masses were often found, which were 1.0–4.1 cm in diameter (mean 2.3cm). Five (35.7%) cases of pancreatic head adenocarcinoma were accompanied by atrophy of the pancreatic body or tail. Ten (71.4%) cases of pancreatic head adenocarcinoma showed dilation of both the bile ducts and pancreatic duct (PD) (Fig 1). The dilated common bile duct (CBD) and PD appeared as a separate double-duct sign, three-segment sign (bile duct and two pancreatic duct segments) or four-segment sign (two bile duct segments and two pancreatic duct segments), according to the site of tumor invasion of the CBD or PD. Three pancreatic head adenocarcinomas showed pancreatic duct dilatation without biliary dilatation, and one pancreatic carcinoma showed dilatation of the bile duct without pancreatic duct dilatation. The distance from the duodenal lumen to the ends of the dilated ducts was 1.1-3.8 cm (mean, 2.2cm). Pancreatic masses were usually hypointense to isointense on T1 weighted images (T1WI) and isointense to mildly hyperintense on T2 weighted images (T2WI). On liver acquisition with volume acceleration (LAVA) images, the masses appeared as poor enhancement, and 3 cases of pancreatic head carcinoma showed slightly enhancement.

***Distal common bile duct carcinoma***

Of the 12 patients with distal CBD carcinoma, 8 (66.7%) had irregular bile ductal wall thickening and one had an intraductal polypoid mass. Three cases (25%) presented both irregular bile ductal wall thickening and an intraductal polypoid mass (Fig 2). CBD dilation was found in 10 patients (83.3%). The CBD and PD dilated in two patients, in whom tumor extension to the ampulla and pancreas respectively, were proved pathologically. The dilated CBD and PD of the CBD carcinoma with infiltrated pancreas appeared as a three-segment sign (the proximal and distal segments of the bile duct, and the main pancreatic duct). The shapes of the distal margin of the bile ducts demonstrated blunted, beak, or rat-tail forms. The typical characteristic of cholangiocarcinoma was that the lesion was enhanced in the delayed phase. Four cases showed lesions that were also enhanced in the arterial and venous phase, because the tumors were rich in fibrous tissue and the peripheral bile ducts had been infiltrated by inflammatory cells.

***Ampullary carcinoma***

Of the 10 patients with ampullary carcinoma, 7(70%) had an irregular nodular mass at the distal margin of the pancreaticobiliary junction (Fig 3) and 4 cases of ampullary mass bulged into the duodenum. No discernible mass was evident but irregular periductal thickening and bile duct wall stenosis was seen in three cases. Nodular masses were 0.5–1.8 cm in diameter (mean, 1.1 cm). Eight (80%) cases of ampullary carcinoma showed dilation of both the bile ducts and PD (Fig 3). The dilated CBD and PD appeared as a double-duct sign that was different from that of pancreatic adenocarcinoma. The distance from the duodenal lumen to the ends of the dilated ducts was 1.7-8.6 mm (mean, 4.4 mm) that was shorter than that of pancreatic carcinoma. Two cases showed biliary dilatation without pancreatic duct dilatation. The lesions presented hypointense to isointense on T1WI, slightly hyperintense on T2WI and mild to moderate enhancement in the venous phase.

***Periampullary duodenal carcinoma***

Of the 5 patients with periampullary duodenal carcinoma, 3(60%) had polypoid mass (Fig 4) and 2 had eccentric irregular duodenal wall thickening and luminal narrowing. In three patients, both the biliary and pancreatic ducts dilated. Two patients showed moderate dilatation of the bile duct without pancreatic duct dilatation. The lesions presented hypointense to isointense on T1WI, hyperintense on T2WI and moderate to obvious enhancement in venous phase.

***Distal common bile duct inflammatory stenosis***

Some cases of carcinoma in our study were misdiagnosed as inflammatory lesions. Figure 5 shows a 78-year-old female with distal common bile duct inflammatory stenosis. Whenever eating greasy food, she felt right upper quadrant pain. At the following third, sixth and twelfth month follow-up review, the lesion did not change and the tumor markers were normal. Distal common bile duct inflammatory stenosis is gradual and the incrassate bile ductal walls often show linear homogeneous enhancement in the arterial phase. Mild or moderate dilatation of the biliary tree could be found without the PD dilatation.
